# Physiological outcomes from mind-body resiliency programs in healthcare workers: A scoping review

**DOI:** 10.1371/journal.pmen.0000332

**Published:** 2025-05-23

**Authors:** Jamie Kronenberg, Justin J. Merrigan, Catherine Quatman-Yates, Angela Emerson, Morgan Orr, Riley Summers, Joshua A. Hagen, Maryanna Klatt

**Affiliations:** 1 Human Performance Collaborative, Office of Research, The Ohio State University, Columbus, Ohio, United States of America; 2 School of Health and Rehabilitation Sciences, Division of Physical Therapy, The Ohio State University, Columbus, Ohio, United States of America; 3 Center for Integrative Health, Department of Family and Community Medicine, College of Medicine, The Ohio State University, Columbus, Ohio, United States of America; 4 Department of Integrated Systems Engineering, The Ohio State University, Columbus, Ohio, United States of America; 5 Gabbe Wellbeing Office, Ohio State University Wexner Medical Center, The Ohio State University, Columbus, Ohio, United States of America; PLOS: Public Library of Science, UNITED KINGDOM OF GREAT BRITAIN AND NORTHERN IRELAND

## Abstract

Mind-body resiliency programs have improved perceived resiliency and stress in healthcare workers but less is known about physiological impacts. This scoping review aims to evaluate current methodologies and physiological outcomes of different mind-body programs in healthcare settings. The initial literature search revealed 19457 studies across seven databases (PubMed, Embase, Cochrane Library, Web of Science, Scopus, PsychInfo, and CINAHL) from inception through 8/6/2024. Forty-one studies met the inclusion criteria of peer-reviewed original research studying the effects of mind-body programs (i.e., Mindfulness Based Stress Reduction, yoga, meditation, breathwork, biofeedback) on physiological measures (i.e., blood pressure, heart rate, respiration rate, heart rate variability, sleep) in healthcare workers. Two reviewers independently extracted data from each included study into condensed tables and assessed trends in study design, methodological processes, and physiological outcomes. Conflicts exist in balancing the high cost and validity of clinical apparatuses with more cost effective and user-friendly means of assessing physiological measures within real-world healthcare settings. Most within session investigations found positive impacts of mind-body programs on immediate physiological outcomes, which is expected considering the common theme to induce parasympathetic states. Programs of ≤6 weeks appeared more effective at inducing physiological improvements in healthcare workers currently experiencing high stress or impaired resting physiology. Longer mind-body programs (8–12 weeks) generally improved resting heart rate and blood pressure while having inconsistent effects on heart rate variability. Some investigations identified engagement in more mind-body activities resulted in greater physiological improvements. Discrepancies in findings may pertain to variations in population descriptions, mind-body intervention requirements, and methodology of physiological recordings. Future work should recruit multiple groups with varying stress levels and controls, implement interventions geared towards the time requirements of healthcare workers, and utilize validated physiological recordings at adequate time points throughout and beyond the intervention to determine the trajectory of long-term physiological adaptations.

## Introduction

Chronic accumulations of improperly managed workplace stressors in healthcare settings increase the risk of burnout [1] and consequences to physical health (e.g., hypercholesterolemia, cardiovascular disease, prolonged pain and fatigue), psychological health (e.g., insomnia, depression, anxiety, suicidal ideations), and occupational performances (e.g., dissatisfaction, absenteeism, employee retention, patient care) [2]. Exposure to repeated or chronic environmental stressors, lack of adaptation, and an inability to shut off the stress response can lead to maladaptive neural and neuroendocrine responses, which may contribute to long-term health deterioration [3–5].

Specifically, activation of the sympathetic and deactivation of the parasympathetic nervous systems occur, which corresponds with heighted states of arousal and impaired states of relaxation [6,7]. The sympathetic nervous system response to stress includes increased respiration rates, heart rates and blood pressure to help deliver vital energy substrates (i.e., blood glucose, amino acids, free fatty acids) and oxygen to the body [8]. To balance the stress response, the parasympathetic nervous system can act as an antagonist by leveraging controlled respiration and heart rates [8]. Thus, levels of stress and relaxation may be identified via cardiorespiratory biofeedback of blood pressure, respiration rates, heart rates, and heart rate variability [3].

The baroreflex is a feedback system that triggers withdrawal of sympathetic activation including decreased heart rate, blood pressure, and vasodilation to return to homeostasis while the respiratory sinus arrhythmia (RSA) describes the speeding up (during inhalations) and slowing down of the heart rate (during exhalations) by the vagus nerve due to respiration changes [9,10]. Together the baroreflex and RSA describe the interplay among respiratory and cardiovascular systems on modulating the autonomic nervous system. In the presence of acute or chronic stress (sympathetic overload), an individual may experience an increase in heart rate [11], blood pressure [12,13], and respiration rate as breaths per minute [14,15], as well as lower heart rate variability (HRV) defined as a decrease in variation of time between heart beats [6,11,16,17]. Acute stress-related increases in physiological parameters may persist due to distorted healthy lifestyle habits (i.e., poor sleep) or prolonged distress which prevents individuals from returning to a healthy baseline [18]. Thus, inadequate sleep can both be caused by and contribute to stress and poor stress management behaviors [19,20].

One increasingly popular method for managing stress is implementation of mindfulness based interventions (MBIs), which have demonstrated positive impact regarding perceived levels of mindfulness, resiliency, and sleep quality in healthcare settings [21–23]. Bringing awareness to one’s breath, such as slow controlled breathing or counting natural exhalations [24], is a primary aspect of many mindfulness techniques which subsequently impacts respiration rate and modulates parasympathetic dominance during and after environmental challenges [14]. With mounting evidence demonstrating that mental and physical health are inextricably linked [11], the perceived increase in resiliency and decrease in stress from mind-body programs in healthcare settings would suggest concomitant changes to physiological parameters. Yet, uncertainty remains as to the impact of mind-body programs on various physiological parameters (Table 1) particularly in healthcare settings. Therefore, the purpose of this scoping review was to gain insight into the current methodologies being used to implement mind-body programs (Table 2) and assess their impact on physiological measures in healthcare workers to help identify gaps in the existing research and inform future programs geared towards promoting mind-body resiliency in healthcare workers.

**Table 1:** Physiological metric definitions.

Metric	Acronym	Definition
Blood pressure	BP	Force of blood against artery walls during heart contraction and relaxation.
Systolic BP	SBP	Pressure inside arteries during heart contraction to pump blood to the body.
Diastolic BP	DBP	Pressure inside the artery when the heart is at rest and is filling with blood.
Heart Rate	HR	Number of heart beats per minute. Measured by durations of R-R intervals.
Pulse Rate	PR	A measure of heart rate via expansion and contraction of arteries.
Respiration Rate	RR	Number of complete breaths per minute.
Heart Rate Variability	HRV	Fluctuation in the time intervals between adjacent heartbeats, measured via electrocardiography (ECG) as time between successive R peaks in ECG.
Pulse Rate Variability	PRV	Fluctuation in the time intervals between adjacent heartbeats, measured from blood flow changes via photoplethysmography as time between peaks in pulse waves.
HRV- Standard deviation of N-N interval	SDNN	Represents sympathetic and parasympathetic input [6], but primarily parasympathetic during slowed breathing [16]
HRV - Root mean square of successive differences of R-R	RMSSD	Reflects vagal modulation via parasympathetic activity with little influence of respiration rates [25,26]
HRV - Percent of N-N that differ by > 50ms	pNN50	Correlates with parasympathetic activity, RMSSD, and HF power [16]
HRV – Low frequency (0.04–0.15 Hz) power	LF	Reflection of baroreflex activity rather than cardiac sympathetic innervation [27,28]. Slow breathing (< 9 breaths/ min) increases LF [29].
HRV - High frequency (0.15–0.4 Hz) power	HF	Reflects parasympathetic respiration activity (9–24 breaths per minute) [6,16,30]. Low HF power is related with stressful and anxious states [16].
HRV - LF to HF power	LF/HF	Ratio of LF and HF power, reflects sympathetic/ parasympathetic balance.
Sleep		Typically assessed as a duration of time asleep or perceived sleep quality.
Respiratory Sinus Arrhythmia	RSA	The speeding up (during inhalations) and slowing down of the heart rate (during exhalations) by the vagus nerve due to respiration changes.

**Table 2:** Mind-body resiliency program definitions.

Intervention	Description
**Mindfulness Based Stress Reduction (MBSR)**	The original program developed by Dr. Jon Kabat-Zinn or any study based on this original design that included a 2.5 hour/week, 8 week course with a 1 day retreat focused on formal mindfulness meditation techniques.
**Yoga**	Any form of Yogic practice, such as restorative yoga, Kripalu, Hatha, Kriya, Pranayama, Raja, and Suryanamaskar
**Meditation**	Transcendental and mantra-based meditations or other guided meditation programs including neuro-meditation or yogic meditation.
**Breathwork**	Guided yogic breathwork, focused nose or rhythmic breathing, and dialectal behavior therapy
**Biofeedback**	Providing physiological data as feedback to reinforce self-awareness of breath, positive emotion, positive physiological change, muscle relaxation
**Other Mind-Body Programs**	Various stress management techniques aside from the main identified themes, such as sound therapy, mindful moments, stress management strategy and mindfulness teachings

*Many studies included a combination of interventions such as yoga AND breathwork.

## Methods

This scoping review aims to inform methodologies used to assess the impact of mind-body resiliency programs emphasizing mindfulness techniques on physiological measures in healthcare workers. The Preferred Reporting Items for Systematic Reviews and Meta-Analyses (PRISMA-ScR) scoping review extension checklist (S1 Checklist) was used to guide this scoping review [31], as well as input from professionals and previous literature [32,33].

### Data sources and search

In consultation with a health sciences librarian, the research team developed an a-priori search strategy and scoped the literature across seven databases (PubMed, Embase, Cochrane Library, Web of Science, Scopus, PsychInfo, and CINAHL) from inception through 8/6/2024. In summary, search terms from the title, abstract, or keywords included “Mindfulness” OR up to 50 other synonyms AND “Healthcare Provider” OR any relevant synonyms and health care occupations AND “Physiological” OR any relevant synonymous metrics. An example of search terms used for the PubMed search are included in Table 3, while the complete search terms are available from authors upon request. The initial search yielded 19457 articles, which were uploaded to a systematic review software (Covidence, Melbourne, Australia) to manage and review the articles.

**Table 3:** Example of search terms used in PubMed for the current scoping review.

Theme	Keywords
**Mindfulness**	Mindfulness, self-compassion, Meditation, meditate*, Relax(ation) therapy/program/intervention/practice/exercise/technique, Autogenic, Breathing exercise(s)/intervention(s), breathwork, breath work, breath based, paced respiration, imagery, yoga, gentle movement, Biofeedback, Integrative Medicine, Gratitude
**Physiological Effects**	Vital signs, blood pressure, hypertension, Heart/cardiac rate/rhythm/coherence, pulse, heartbeat, Respiratory/respiration/breathing/breath rate(s) Parasympathetic, sympathetic, autonomic, Biofeedback
**Healthcare Workers**	Health Personnel + , physician, doctor, nurse, physician assistant, nurse practitioner, physical therapist, occupational therapist, respiratory therapist, speech language pathologist, dietician, phlebotomist, pharmacist, social worker, case manager, psychologist, counselor, healthcare administrator, medical assistant, patient care associate, certified nursing assistant, emergency medical technician, paramedic

### Study selection: inclusion criteria

Studies included in this scoping review were limited to English-language only, peer-reviewed original research and were assessing the effects of a mind-body program on physiological measures in healthcare workers. Specifically, we included experimental (randomized controlled trials (RCT), quasi-randomized controlled trials, non-randomized controlled clinical trials), quasi-experimental (within pre/post studies, interrupted time series), and observational (cohort, case control, cross-sectional) original research study designs. Eligible studies included healthcare workers, which were defined as employees or contractors, or clinical students that are currently regularly providing in-person services at a healthcare facility. The focus of the review was on healthcare workers; however, students of healthcare programs were included if they were described as senior or graduate levels with patient-care responsibilities in healthcare facilities. Eligible studies must have implemented some form of structured mind-body program with mindfulness or relaxation techniques that aim to alter healthcare workers’ physiological or perceptual levels of stress and relaxation. The studies generally sought long-term outcomes from mind-body programs (Table 2) but considering the healthcare worker environments we also included short-term outcomes to mind-body sessions. We included variations of the main physiological metrics found in Table 1. If multiple publications appeared to describe the same study design and data, we included the most recent.

### Study selection: screening

All personnel were trained and provided with a checklist of eligibility criteria to help ensure consistency amongst reviewers. Additionally, all study titles and abstracts were independently screened by at least two individuals including at least one senior reviewer for full text screening. All conflicts were resolved by discussion until mutual agreement was reached within the group of senior reviewers. Studies that met all inclusion and exclusion criteria following full text screening were included in this analysis.

### Data extraction and quality assessment

Two reviewers independently extracted data from each included study. Data were entered into a standardized data extraction form in Microsoft Excel (Microsoft Corp, Redmond, WA). Extracted information included: citation, year, country, study design, healthcare setting, healthcare worker population, sample size, mind-body intervention, comparison or control group, physiological metric methods and analysis, reported results, and limitations. All conflicts were resolved through discussion.

### Data synthesis and analysis

Two researchers (JK and JJM) carried out the initial analysis of the extracted data to assess trends in study design, methodological processes, and overall physiological outcomes after a mind-body program. The interventions included herein were categorized based on their attributes as: 1) mindfulness-based interventions or Mindfulness-Based Stress Reduction, 2) yoga or meditation, 3) biofeedback or Attention-Based Training. The protocols were also compared within the themes of having investigated: 1) acute interventions and outcomes, 2) short term interventions and outcomes (≤ 6 weeks), 3) long term interventions and outcomes (~8–12 weeks). Discrepancies in coding were discussed amongst the research team to achieve consensus.

## Results

### Literature search and selection

**Fig 1:**
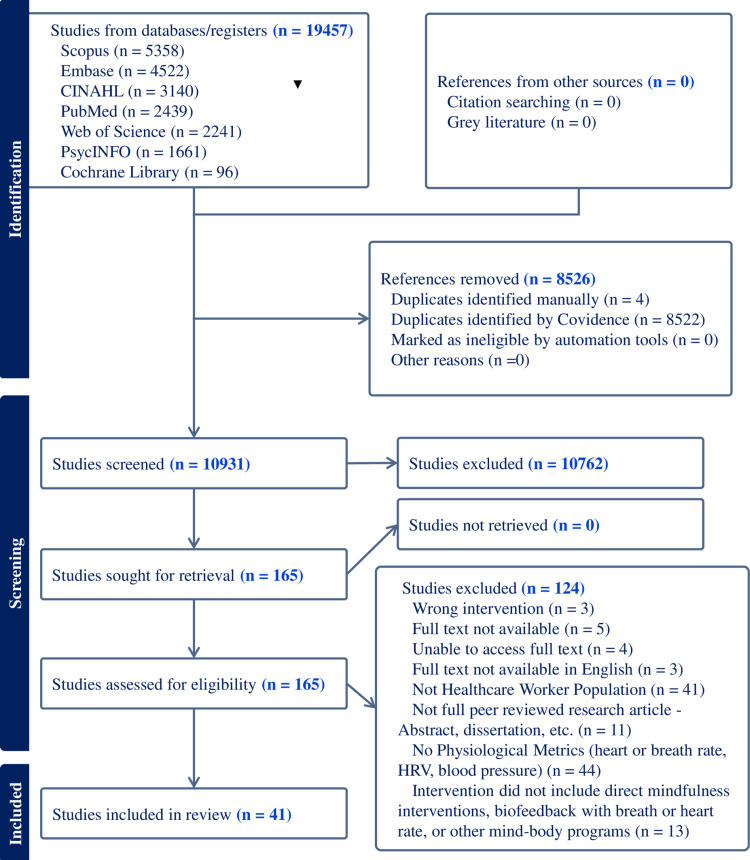
PRISMA flow diagram for study selection.

The initial search from the databases yielded 19457 articles, 8526 of which were removed as duplications. Of the 10931 articles screened, 165 were included for full text review. Forty-one studies that met the inclusion criteria were included in this scoping review for data extraction (Fig 1).

### Study and sample characteristics

All studies were published after 2011. Most studies included in this review were completed in Asia (n = 17) and North America (n = 14) with others completed in Europe (n = 6), Oceania (n = 2) and South America (n = 2). Twenty-one of the studies included were RCTs, 12 were cohort studies, 2 were cross-sectional studies and 6 were quasi-experimental. Most studies were conducted in hospital settings (n = 27), followed by emergency medicine (n = 4), assisted living/elderly care (n = 3), outpatient care (n = 2), intensive care (n = 2), psychiatric facility (n = 2), oncology (n = 1), and cardiac/respiratory care (n = 1) with some studies being conducted in multiple settings (n = 3) (i.e., public and private practices). Healthcare professions included physicians, surgeons, residents, nurses, respiratory therapists, physical therapists, social workers, mental health providers, nutritionists, dentists, professional caregivers, and healthcare office staff. Studies sample sizes ranged from 12 to 275 participants. A summary of participant characteristics is described in Table 4.

**Table 4:** Population characteristics.

Reference	Country	Recruitment Setting	Occupation	N	Study Design
[34]	Spain	Public or private practice	Physicians	42	Quasi-Experimental Longitudinal
[21]	Spain	Public or private practice	Physicians	42	Quasi-Experimental Longitudinal
[35]	Germany	Hospital, elderly or ambulatory care center	Nursing staff and office workers	101	RCT
[1]	USA	Emergency medicine	Nurses and physicians	31	RCT
[36]	India	Rural hospital	Female doctors, nurses	57	Cross-sectional
[37]	USA	Hospitals	Internal medicine residents	23	RCT
[38]	India	Ageing and Alzheimer’s center	Professional caregivers	30	RCT
[39]	Ireland	Emergency medicine	Multidisciplinary	39	RCT
[22]	USA	NICU and newborn nursery	Nurses, respiratory therapists	94	RCT
[23]	Brazil	Tertiary hospital at federal university	Pediatric healthcare workers	64	RCT
[40]	France	Hospitals and assisted living facilities	Nurses	45	RCT
[41]	USA	Community based hospital system	Nurses, therapists, physicians	78	RCT
[42]	Taiwan	Psychiatric ward	Nurses	135	Quasi- Experimental
[43]	India	Cardiorespiratory department	Medical Professionals	30	Quasi- experimental
[24]	USA	Hospital	Intensive care unit	34	Cohort Study
[44]	Canada	Tertiary care hospital	Physicians	40	RCT
[45]	Taiwan	Teaching hospital	Mental health provider	60	RCT
[46]	USA	Children’s hospital	Respiratory therapists	64	cohort study
[47]	India	Tertiary care hospital	Nurses	51	RCT
[48]	Japan	Hospital	Nurses	20	Randomized Crossover Trial
[49]	Japan	University hospital	Healthcare Workers	13	Cohort study
[50]	USA	Emergency Room	Healthcare Workers	32	RCT
[51]	India	Intensive Care Units	Residents, physicians, nurses	41	Cross-Sectional
[52]	USA	Outpatient oncology unit	Nurses	12	Cohort Study
[53]	India	Emergency Services	ICU and casualty unit healthcare workers	35	Cohort Study
[54]	India	Hospital Extension	Not specified	22	RCT
[55]	USA	Mental Health Center	Mental health provider	28	Cohort Study
[56]	India	Tertiary Care Hospital	Female nurses	30	RCT
[57]	Australia	Hospital	Junior doctors	18	Cohort Study
[58]	Australia	Hospital	Surgeon	16	RCT
[59]	Jordan	Hospital	Nurses	123	RCT
[60]	India	Tertiary care hospital	Nurses	60	RCT
[61]	Spain	Tertiary care hospital	Clinicians and nurses	21	Cohort Study
[62]	Brazil	Public university hospital	Nurses	115	RCT
[63]	USA	Hospital	Healthcare workers	23	Cohort study
[64]	USA	Public university hospital	Healthcare workers	66	Quasi-experimental
[65]	USA	Public university hospital	Healthcare workers	275	Quasi-experimental
[66]	India	Hospital	Frontline healthcare workers	80	Cohort study
[67]	Japan	Hospital	Nurses	20	Cohort study
[68]	USA	Pediatric tertiary care hospital	Nurses	150	RCT
[69]	India	Tertiary care hospital	Nurses	88	RCT

### Mind-body resiliency programs

Programs in this scoping review included yoga (n = 12), mindfulness-based stress reduction programs (n = 8), meditation (n = 6), breath work (n = 4), biofeedback (n = 4), and other mindfulness-based interventions (n = 7). Programs ranged from short-term immediate outcomes (n = 4), 6- or fewer week programs (n = 15), and 8- to 12-week programs (n = 22). See Table 5 for further details regarding each mind-body program type.

**Table 5:** Summary of study designs and outcomes for mind-body resiliency programs.

Reference	Program Type	Intervention Description	Comparison Group	Program Length	Physiological Measures	Results
[34]	MBSR	8 weekly 2.5 hr sessions, 1 additional 8 hr session.	None	8 weeks	HR via Digital sphygmomanometer (model M3, Omron)	HR ↓ @ Post- and 10-month follow-up
[21]	MBSR	8 weekly 2.5 hr sessions, daily mindfulness exercises with 45 min CDs.	None	8 weeks	BP, HR via Digital sphygmomanometer (model M3, Omron)	BP ↓ ; HR ↓ @ Post- and 10-month follow-up
[35]	Mindfulness Based Intervention	Web-based digital stress management (1) + need-orientation (2) + telephone coaching (3). App-based personality specific stress management with biofeedback (4) + health report (5)	Waitlist Control	8 weeks	HRV (SDNN, RMSSD, LF/HF ratio) via Multi-lead ECG (Corvolution CM300)	No change Pre-Post or against CG.
[1]	Other	“The Pause” A mindful “pause” for 60 sec. after a simulation 90–120 min of losing a patient.	Control Group	One Session	HRV (SDNN) via Chest ECG (Zephyr) and HR, BP via Sphygmomanometer (DINAMAP)	HRV ↑ Pre-Post against CG; HR-; SBP-; DBP-
[36]	Progressive Muscle Relaxation	Muscle tensing for 7-10s then relaxing for 15-20s (Jacobson’s protocol) for 20 minutes every	None	3 months	HR: manual; Breath Hold Time (BHT): stopwatch; BP: NS	HR ↓ ; BP ↓ ; BHT ↑ Pre-Post
[37]	Other	12-min mindfulness videos via PITSTOP. Pause; Inhale; Take note of Self & Task; Observe without judgment & focus; Proceed when ready	12 min “Tweak your week” health video	One Session	HR, HRV via Wrist PPG (Empatica E4), ARTiiFACT Software	HR ↓ ; SDNN-; RMSSD-; pNN50-
[38]	Yoga	1-hour integrated yoga session (Asanas, Pranayama, meditate) 6 days/ week	Waitlist Control	1 month	HR, SBP, DBP via Digital sphygmomanometer (Omron HEM 7130); Sleep Quality via PSQI	HR ↓ ; BP ↓ Pre-Post = CG; Sleep Quality↑ Pre-Post > CG
[39]	Meditation	Four 4-hour sessions (focused attention to mantra-based meditations) over 7 weeks. Practice attention to Maranatha phrases 20-min 2x per day.	Waitlist Control	7 weeks	HR, HRV, sleep quality via Wrist PPG (Fitbit Charge 2) bespoke TickerFit app	HR ↓ ; HRV↑ sleep time↑ within TG and between CG
[22]	MBSR	MBSR course (UMASS Amherst). 3 hours/week, 8 hr retreat between class 5 and 7	Control Group	8 weeks	Pulse Rate Variability (PRV) via Finger PPG (Heart Math System)	PRV- within TG and between CG
[23]	Meditation	1 weekly 30-minute yogic meditation class. 1 additional at home practice per week.	Waitlist Control	8 weeks	Sleep, HR via Polysomnography, ECG, respiratory inductance plethysmography (EMBLA S7000) & PSQI	Sleep quality ↑ within TG; PSQI & HR↓ within TG and between CG
[40]	Meditation	2-3 sessions per week. Rebalance Impulse is a non-invasive cognitive stimulation and mindfulness training device based on applied neuroscience. Mindfulness training included sound therapy and coach-guided meditation associated with light stimulations	yes; no neuro-meditation	4 weeks	HR, HRV via PPG (Polar OH1) Kubios Software and Sleep via Actiwatch (Cambridge Neurotechnology Ltd.)	BP ↓ ; RHR ↓ Pre/Post Hypertensive compared to normal and CG. Sleep efficiency ↑ at session 9 RHR ↓ ; HRV↑ at session 10
[41]	Yoga	1 group Hatha and Raja Yoga session per week. At home Yoga/ breath work 3-5x/week.	Waitlist Control	6 weeks	BP via “standard procedures” & Sleep quality via PSQI	BP- & Sleep Quality↑ Pre-Post compared to CG
[42]	Biofeedback Training	Biofeedback training: 1 weekly 60-min session of biofeedback and short meditations Smartphone-delivered: videos of short meditations and real-time biofeedback 1x/week	Waitlist Control	6 weeks	HRV (SDNN, LF, HF), RR via ProComp Infiniti software, electrocardiogram, respiration sensors	RR ↓ Pre/Post within both TG no diff between CG; HRV (SDNN, LF, HF) -
[43]	Yoga	12 Asanas (30 min) of Suryanamaskar every 5 days for 4 weeks	Aerobic Exercise (30 min walking)	4 weeks	RHR via chest ECG (Polar) SBP, DBP via sphygmomanometer	RHR ↓ ; BP ↓ Pre/Post within TG not between CG
[24]	Mindfulness Based Intervention	Modified MBSR (Mindfulness in Motion), 8 weekly 60 min onsite sessions, and 20 min at home sessions.	None	8 weeks	RR via self-counted breaths	RR ↓ Pre/Post Sessions within TG at weeks 1,2,3,5,6,8
[44]	Biofeedback Training	Biofeedback-based stress management 3x/day reinforced rhythmic breathing, emotions, physiological change from stress. Twice weekly support visits for Part 1, repeated unsupported with CG for Part 2	Waitlist Control	4 weeks (Part 1), 4 weeks (Part 2, first for CG)	HR, BP via Digital sphygmomanometer (AMG Medical #106–925)	BP-; HR- within TG at day 28 and 56 and between CG
[45]	Yoga	12 weekly 60 min class (warm up, breathing, meditation, stretching)	Waitlist Control	12 weeks	HRV Monitor V1.89 by Yang Ying Inc.	LF-; HF-; LF/HF↓ within TG and between CG
[46]	Mindfulness Based Intervention	“Essential Breath” and “Being Present” during 2 30 min meetings separated by 4 months	None	Single Sessions	RR via self-counted breaths	RR ↓ Pre/Post Sessions
[47]	Yoga	Structured Yoga via Asana, Pranayama, relaxation techniques. 2 sessions per week.	Waitlist Control	12 weeks	BP via Digital sphygmomanometer Health sense BP100	SBP ↓ Pre/Post within TG and between CG; DBP-
[48]	Yoga	1 hour guided Yoga session	None	4 weeks	BP, PR methods not specified	BP-; PR- Pre/Post Sessions
[49]	Meditation/ Yoga	1-hour weekly sessions (5 min Meditation, 40 min Yoga/ Breath, 15 min Mindfulness)	None	3 months	PR via self-measured methods	PR ↓ Pre/Post first and last sessions, PR- pre/post intervention
[50]	Meditation	Virtual reality-guided meditation: paced breathing exercises for 3–5 minutes with calming music and audio instructions in 3D vs 2D.	Mobile Group Meditation	4 weeks	Mean LF HRV ΔLF-HRV via eVu-TPS heart rate monitor on their index finger	LF HRV = CG ΔLF-HRV > CG within sessions
[51]	Meditation/ Yoga	Online workshop: Sudarshan Kriya Yoga and Pranayama. 4-4-6-2 cadence breathing (inhale, pause, exhale, pause).	NA	4 days (5 nights)	Time of total, light, deep sleep, SDNN, RMSSD LF/HF, HR, RR via Dozee, Ballistocardiography artificial intelligence-powered contactless monitor	Sleep ↑ ; HRV↑ (SDNN, RMSSD), HR ↓ ; RR and LF/HF no change Pre/Post 4-day Intervention
[52]	Meditation	MP3 player preloaded with guided meditations. Guided meditations were 10 minutes for 8 sessions	None	4 weeks	BP, HR via OMRON-7 series. RR via breath observation while obtaining BPs.	SBP↓ in 5/ 8 sessions; DBP↓ in 3/ 8 sessions; HR↓ in 6/ 8 sessions; RR↓ in 2/ 8 sessions
[53]	Yoga/ Meditation	Shavasana, slow rhythmic nose breathing, progressive muscle relaxation after a night-shift sleep deprivation	None	Acute	BP, LF/HF, DPB, mean HR ICNO Polyrite-D, Kubios, sphygmomanometer	BP ↓ ; LF/HF ↓ ; BP ↓ ; HRV↑ (HF, RMSSD, SDNN) Post Sleep Deprivation to Post Shavasana
[54]	Breathwork	Short breathing technique and long duration breathing technique administered morning and evening for 15 days via video calls.	yes; no breath work	15 days	HR, HRV (RMSSD, HF, LF) via ECG Physiograph-D (Recorders Medicare Systems)	HRV- & HR- Pre/Post no difference within TG and between CG
[55]	Yoga-Based Stress Management	Kripalu Yoga with stress management psychoeducation (similar to MBSR).	Cognitive Behavioral Stress Management 1 hour/week	8 weeks	HR, BP methods not specified	HR↓ within TG but not between CG; BP↓ in TG, BP↑ in CG Pre/Post, 2 & 6 month follow-up
[56]	Meditation	Daily 20 min guided Mahamantra Chanting	Waitlist Control	45 day minimum	HRV (RR, SDNN, pNN50, LF, HF, LF/HF ratio) via Multi-lead ECG and Kubios Software	HRV (SDNN)↑; HRV (LF/HF ratio, LF)↓, HR ↓ Pre/Post within TG > CG
[57]	Yoga	1-hour private Hatha yoga sessions per week. 4-hour retreat. 2 hours per week of homework (Breath training, relaxation, meditation)	45-60 min Group Fitness. 1x/wk. 2.5 hours of homework.	8 weeks	SBP, DBP, RHR via Sphygmomanometer (Welch Allyn), HRV (RMSSD and SDNN) via finger PPG Corsense and Elite HRV app.	BP-; RHR-; HRV rMSSD-, HRV SDNN ↑ Pre/Post not different than CG.
[58]	Stress Management Training	Education and rehearsal of stress management strategies, strategic planning, mental rehearsal, relaxation training	Control Group	Single Session	HR, HRV SDNN/NN × 100 via Chest ECG Wireless S801i HR monitor (Polar)	HR-, HRV↑ within TG, CG no changes
[59]	MBSR	2 x 60 min sessions/ week: specific focus on mindful eating, stretching, sitting with awareness of breath, body scan, or thoughts	Control Group	4 weeks	Self-reported sleep duration	No effect on sleep duration between intervention and control groups
[60]	Yoga	Chair yoga 20 minutes a day, 5 days a week	Control Group	2 months	HR, RR interval, SDNN, RMSSD, PNN50, LF, HF, LF/HF ratio via physiopac system	HR ↓ , SDNN ↑ , PNN50 ↑ , LF ↓ , HF ↑ , LF/HF ratio ↓ , all significantly> than control; RMSSD no difference
[61]	Biofeedback	5 weekly, in person sessions of 45–60 min, respiratory, upper trapezius tension, HRV biofeedback	None	5 weeks	HR, SDNN, RMSSD, LF, HF, LF/HF, RR via NeXus-10 MKII device, Biotrace+ software	SDNN ↑ , LF/HF ↑ , RR ↓ were significantly different pre/post
[62]	Biofeedback	Trained on cardiovascular biofeedback technique and software, interactive games: practiced deep breathing guided by a pacer (6 breaths/min, inspiration ratio 50/50, pause after inspiration of 32% and after expiration 20%) lasting 10 min per session, 9 meetings (3 per week over 3 weeks)	Control, completed the jigsaw puzzle with no self monitoring	3 weeks	SDNN, rMSSD, LF/HF, heart coherence via emwave pro plus	LF/HF ↑ , heart coherence ↑ , both were significantly different between groups. There were time-related differences in both groups for SDNN and rMSSD when compared to baseline
[63]	Mindfulness Based Intervention	Virtual training to use mindfulness-based app. Participants completed 5 days of 3-min baseline and post HRV readings. Participants chose breathing speed and duration when doing virtual HRV biofeedback. Mean 26 days with >10-min biofeedback.	None	2 months	HRV, rMSSD, via OptimalHRV	RMSSD ↑ 13% but was not significantly different pre- to post-intervention
[64]	Mindfulness Based Intervention	Mindfulness in Motion: Eight weekly one-hour virtually synchronous group meetings and 10 minutes of mindfulness home practices 3 times per week using a mobile app and wearable sensors monitoring sleep and nocturnal metrics	None	8 weeks	HRV, rMSSD, RR, sleep via Oura Ring	HRV ↓ nights after intervention compared to nights before, no significant improvement in resting physiological metrics
[65]	Mindfulness Based Intervention	Mindfulness in Motion: Eight weekly one-hour virtually synchronous group meetings and 10 minutes of mindfulness home practices 3 times per week	Control group	8 weeks	RR via self-report	RR ↓ after each session. RR ↓ during weeks 3 through week 8 compared to week 1
[66]	Yoga	Structured 1-hour yoga sessions at the hospital three times a week for 12 weeks	None	12 weeks	SBP, DBP, HR (methods not specified)	No significant differences.
[67]	Healing/ Relaxation Therapy	Followed Reiki guidelines for a single 60 minute session: 12 positions, hands-on treatment (knees, ankles, soles), 30 min each front and back body	None	60 min session	PR, RR, BP (methods not specified)	PR and DBP ↓ post Reiki intervention; RR and SBP no diff
[68]	Healing/ Relaxation Therapy	The healing touch intervention group received a 4–7 minute session “Noel’s Mind Clearing” technique found in Healing Beyond Borders	Deep breathing comparison group (same intervention length, read and deep breathing).	4 weeks	HR, BP, RR via sphygmomanometer and researcher visualization of breathing	↓ in RR approached significance post treatment, but was significantly ↓ at follow up compared to control group
[69]	Yoga	Participants were administered supervised Standard Common Yoga Program for Health Professionals	Control group	40-45 minutes, three times per week for a total of 8 weeks	HR, BP, HRV (rMSSD, SDNN, LF, HF) via standard sphygmomanometry, BrainTap-HRV Single Lead ECG	Post Yoga v Control: HRV (LF, HF, TP, NN50, pNN50) ↑ and HR↓ Yoga Group Pre to Post: HRV (RMSSD, NN50, pNN50, LF, HF, TP) ↑ and HR, BP↓

↑: Significant increase in value; ↓ : Significant decrease in value; -: no significant change in value; CG: Control Group; TG: Treatment Group; WASO – wake after sleep onset, SQ – sleep quality; PSQI - Pittsburgh Sleep Quality Index; BP - Blood pressure;SBP - Systolic BP; DBP - Diastolic BP; HR - Heart Rate; PR - Pulse Rate; RR - Respiration Rate; HRV - Heart Rate Variability SDNN- Standard deviation of N-N interval; RMSSD - Root mean square of successive differences of R-R; pNN50 - Percent of N-N that differ by > 50ms; LF – Low frequency (0.04–0.15 Hz) power; HF - High frequency (0.15–0.4 Hz) power; LF/HF - LF to HF power.

### Physiological measures and technology

Studies included in this review investigated heart or pulse rate (n = 27), heart rate variability (n = 20), blood pressure (n = 18), respiration (n = 7) and sleep (n = 3). Several approaches were used to measure physiological outcomes with themes of clinical or research grade devices (n = 29), commercially available wearable or contactless technology (n = 10), and practical device free options (n = 8). Blood pressure was measured by a digital or manual sphygmomanometer (blood pressure cuff) (n = 11), which was presumed to also be the case in studies that didn’t specify a method for blood pressure (n = 4). Resting heart rate, also referred to as pulse rate, was also measured with a digital blood pressure cuff (n = 5 + 2 not specified), multi-lead electrocardiography (ECG, n = 5), single-lead or chest strap ECG (n = 4), manual measure (n = 2), photoplethysmography (PPG) wearable technology (n = 4), finger probe PPG (n = 3), and by other non-specified means (n = 4). Heart rate variability or pulse rate variability was measured by multi-lead ECG (n = 6), single-lead or chest strap ECG (n = 5), PPG wearable technology (n = 4), finger probe PPG (n = 4), and Ballistocardiography (n = 1). Specific software cited for HRV analysis included Heart Math System, ProComp Infiniti TM, Kubios, BrainTap HRV, OptimalHRV, EmWare Pro Plus and ARTiiFACT. Respiration rate was primarily collected using self-counting exhalations (n = 3), observer counting inhalations (n = 1), a stopwatch for breath hold time (n = 1), and respiratory inductance plethysmography (n = 2). Sleep was measured using a mixture of devices including polysomnography, electroencephalogram, electrooculogram, ballistocardiography and wearable technology (n = 5), as well as Pittsburgh Sleep Quality Index for subjective measures of sleep (n = 3).

### Physiological outcomes

Several studies found significant decreases in SBP and DBP following mind-body programs (n = 7), while a few studies noted no change to BP within the treatment group or between the control group (n = 4). Other studies found a decrease in only DBP [40] or only SBP [47]. Several studies found mind-body practices to lower HR or PR (n = 12), of which two found the decreases in HR to be greater than the control group, while other studies did not find significant HR changes following mind-body programs (n = 4). Several studies found significant differences in SDNN [51,53,56,61,62], RMSSD [51,53,62,63], pNN50 [53,60], LF [53,56,60], HF [39,60], LF/HF [45,53,56,60–62] and general improvements in HRV [1,50,58,63,69]. Others found no change in HRV within treatment groups or between control groups [22,35,54,57]. Specifically, no significant differences were found in SDNN [37,42], RMSSD [37], pNN50 [37], LF [42,45], or HF [42,45]. Studies that assessed respiratory rate found significant decreases in respiratory rate [24,42,46,52,61,65]. Similarly, sleep quality was improved from mind-body programs [39,51], as well as increased total sleep, deep sleep, REM sleep and light sleep durations [51]. However, one study reported no effect of mind-body programs on sleep duration[59]. Only one study reported negative effects of mind-body programs on HRV, however authors cited lack of adjustment for potential influential and impactful covariates for HRV [64]. All study outcomes are summarized in Table 5.

### Reported limitations by included studies

The most frequently cited limitation was a small or homogeneous sample size [1,21,34–41,43,45–50,52,53,56–58,61,63,69]. Conversely, sample heterogeneity increased difficulty of interpreting results [22]. Several studies lacked a control group [21,34,36,40,46,47,51,55,61,63,67] and identified differences between groups at baseline [37,44,52]. Research team and treatment group blinding was also cited as a limitation [40,48,52]. Several studies noted that follow up testing would have improved the strength of their findings [22,23,38–41,43,46,50,51,53,56]. Additionally, while some studies noted there was not enough structure to their intervention [42,52,58] or limits in scope [48,49], others reported overly high expectations of their participants during the intervention [35,39]. Logistical limitations included technological difficulties with physiological measurements [22,35,51], institutional support [24], space constraints [45], and disruption from COVID-19 [62,63,69].

## Discussion

The search strategy performed for this scoping review yielded 41 articles that assessed physiological outcomes in healthcare workers following mind-body resiliency building programs with emphasis on mindfulness. The studies included a diverse array of healthcare populations from different regions of the world, mind-body resiliency building program delivery, and physiological measurement methodology and outcomes. The studies included research from 12 countries spanning five continents. Fourteen different healthcare professions were represented in eight different health care settings. There was great variety in the structure and length of mind-body resiliency programs, which were evaluated using various techniques to capture physiological changes. A mixture of clinical and non-clinical wearable technology or self-assessment was used to measure physiological outcomes. Although differences existed amongst the studies, most mind-body resiliency programs had a positive impact on physiological metrics in healthcare workers.

### Physiological considerations

Most health care workers in this review reported high levels of perceived stress [1,21,22,34,38,39,41,42,44,45,47,48,50,57] or depression [36] prior to beginning their mind-body resiliency program. Prior presence of reported stress may impact baseline physiology and response to mindfulness techniques. For example, rapid respiration and heart rates and low HRV are associated with stress and anxiety [6,11,16,70] but can be improved by mindfulness techniques to levels associated with relaxed mind-body states [71]. Thus, leveraging controlled rhythmic and attentive breathing during mind-body resiliency programs can offset the stress response with more RSA mediated PNS modulation [9,72–74]. Therefore, individuals with worse stress impaired physiological states may be more likely to experience positive detectable improvements in physiological metrics.

There was a great amount of variation in the tools used to assess physiological parameters in the current review, particularly for analyzing inter-beat intervals (R-R intervals) to capture HR and HRV. Several studies utilized multi-lead ECG [35,42,53,54,56], which is a widely accepted gold-standard tool for physiological measurement in research and clinical settings. Research-oriented criterion devices are often more difficult to utilize and wear in non-clinical settings as they include wired electrode sensors which are generally more expensive and burdensome to the participant and researcher [75]. Single-lead [69] chest strap form factor [1,43] ECG devices used in the current review may provide valid recordings with greater ease of use [76,77]. Yet, ECG recordings often require additional software, such as Kubios [40,53,56], ARTiiFACT [37], Elite HRV [57], Biotrace+[61], or Emwave Pro Plus [62], for data analysis and interpretation. Photoplethysmography (PPG) estimates heart rate from contraction-induced fluctuations in blood volume via light reflections in small devices worn on the wrist [37,39,58], finger [22,50,57], or arm [40] in the current review. Concerns with PPG devices often include their validity, but the error from PPG devices compared to ECG may be acceptable when considering their improved practicality and compliance [78].

Ultimately, many methods have been used to capture physiological states during mindfulness interventions which makes it difficult to decipher comparisons across previous findings. Practitioners and researchers must consider the balance of cost, simplicity, and validity of the methods being employed to increase compliance and confidence of acquired physiological recordings. Although multi-lead ECG were used in clinical studies herein, practitioners and researchers may consider the use of validated single-lead ECG devices that improve accessibility and ease of data collection in field settings. Some single-lead ECG, PPG, or self-monitoring methods also permit data collection remotely in real-world environments to potentially capture lasting effects of mind-body programs. Participants may also experience greater self-awareness by receiving biofeedback. Thus, data collection methods, such as single lead ECG, may help inform physiological responses to mind-body programs while also enhancing individuals’ self-awareness and reflective practices. Immediate physiological outcomes to mind-body program sessions

Generally, short-term effects of mind-body program sessions revealed positive impacts on physiological outcomes. Two studies found slower self-counted breath rates after mindfulness sessions compared to the start of each session [24,46,61,65]. These findings are expected for sessions that intentionally control breathing rates [65,79,80], which would theoretically yield lower HR and altered HRV according to the RSA [10]. One-hour of five restorative yoga poses and deep breathing non-significantly slowed HR (73 bpm to 64 bpm) [48], while Shavasana Yoga after a night of sleep deprivation slowed HR and improved HRV (SDNN, RMSSD, LF, HF, LF/HF) [53]. During healthcare simulations, a short mindful moment [1], general stress management training [58] improved HRV (e.g., higher SDNN) but did not affect HR or BP. Conversely, a short mindful activity (PITSTOP) resulted in slower HR but unchanged HRV (SDNN, RMSSD, pNN50) [37]. Yet, participants had improved PRV the nights following Mindfulness in Motion sessions compared to the remaining 6 nights of each week [64]. Others found simultaneous changes to BP, HR, and respiratory rate when nurses completed guided meditations [52]. More experience with virtual reality guided meditations and other forms of meditation was correlated with greater improvements in LF HRV and more relaxed states [50,61,62,68]. Thus, a variety of mind-body techniques seem efficacious for improving physiological states in the short-term, but changes to physiological metrics may not coincide and the current physiological state of participants and their mind-body experience may impact results.

### Mindfulness programs of ≤ 6 weeks

Sleep quality generally improved after yoga programs lasting 4–6 weeks [38,41] while control groups noted worsened sleep quality in conjunction with increased stress, depression, and anxiety [38]. However, one study found no effect of a 4 week MBSR program on self-reported sleep [59]. Healthy healthcare workers had no change in BP after completing 6 weeks of one group yoga session and 3–5 home yoga sessions per week compared to the control group [41]. Moderately stressed medical professionals had reduced HR and BP following 4 weeks of Suryanamaskar Yoga (12 Asanas, ~ 30 min, every 5 days), but the changes were no different than aerobic exercise (30 min of treadmill walking at 40–60% of maximal heart rate) [43]. Four weeks of 60-minute integrated yoga sessions 6x per week in caregivers resulted in lower HR and BP, but the post intervention values were not different than the waitlist control group [38]. Unfortunately, the study mentioned did not directly examine an interaction effect of group and time, and the yoga group had an 11% greater decrease in HR compared to the control group [38]. For short yoga interventions, it appears that changes in HR and BP are less likely in healthcare workers currently experiencing little stress [41] compared to moderate or increasing stress [38,43].

Similarly, nurses with hypertension had lower BP and HR after 4 weeks of a neuroscience driven mindfulness training with sound therapy and guided meditations 2-3x per week compared to nurses with normotensive BP and control groups [40]. Heart rate, HRV, and sleep efficiency were also improved more than the control group [40]. Female nurses that completed daily Mahamantra chanting for at least 45 days experienced increased SDNN HRV and decreased LF/HF ratio, LF, and HR more than the control group [56]. Nurses who documented having negative mental health symptoms had slower respiratory rates, but unaltered HRV (SDNN, LF, HF), after completing 6 weeks of biofeedback training compared to controls [42]. Using a stress management tool three times per day for 28 days, which reinforced rhythmic breathing, positive emotions, and biofeedback, did not alter BP or HR in normotensive physicians [44]. Short breathing (21 powerful cycles of breath followed by 30 + second breath holds) and long duration breathing techniques administered morning and evening for 15 days did not alter HR or HRV (RMSSD, HF, LF) [54]. Therefore, short term meditation, biofeedback, or breathwork programs also seem more efficacious in improving physiological outcomes in healthcare workers currently experiencing unmanageable amounts of stress or impaired resting physiology.

### Mindfulness programs of 8–12 weeks

Several studies investigated the MBSR intervention, which is a structured psycho-educational intervention that employs mindfulness and meditation practices aimed to improve the mind-body response to stressors [64,65,81–83]. Traditional MBSR protocols require an orientation session, eight weekly 2.5- to 3-hour classes, and a 7- to 8-hour retreat between week 6 and 7. Two studies followed typical MBSR protocols, including 45-minute daily practices, then provided a 10-week maintenance protocol which included one 2.5-hour session every month. Physicians in both studies had decreased HR and BP after the 8-week MBSR which was maintained throughout the 10-month maintenance period and associated with states of perceived relaxation [21,34]. Interestingly, a greater number of recorded hours of home practice was related with greater reductions in BP [21]. When comparing an 8-week Kripalu Yoga with MBSR-based psychoeducation program to cognitive behavioral stress management, the protocols were equally as effective at reducing HR and BP [55]. Therefore, these long-term stress management programs including mindfulness training seem to positively impact BP and HR.

Conversely, PRV did not change and was not correlated with reductions in perceived stress after Newborn Nursery and Neonatal Intensive Care Unit (NICU) healthcare workers completed MBSR [22]. These results may have been due to using the Heart Math finger probe system, the length of measurement (5-minute measure after a 5-minute rest), or a relative insensitivity of PRV for detecting biologic implications for reduced stress [22]. Similarly, the Mindfulness in Motion intervention has shown reductions in self-reported respiration rates [65] but no change in nocturnal resting PRV via the OURA ring throughout the 8 week program [64]. Yet, variations of 8-week web- or application-based stress management interventions with and without biofeedback were not effective at improving HRV (SDNN, RMSSD, LF/HF ratio) measured by clinical ECG [35] or OptimalHRV [63]. The lack of findings in the aforementioned study could have been impacted by the 74% dropout rate due to COVID-19 and technological difficulties [35] as well as lack of adjustment for factors that would negatively impact HRV [64]. Blood pressure, HR, and HRV (RMSSD) remained unchanged following Yoga interventions or compared to control groups [57,66], while other structured Yoga programs demonstrated significant improvement to BP and HRV metrics compared to control conditions [69]. Thus, there seems to be a lack of evidence that HRV can improve or be capable of detecting physiological improvements from 8-week mindfulness programs.

An 8-week yogic meditation program, including one weekly class and one at-home practice, resulted in improved sleep quality, as well as decreased HR compared to the control group [23]. Only SBP was improved more than the control group after 12 weeks of two structured yoga (Asana, Pranayama, relaxation techniques) sessions per week [47]. Implementing short (20 min) daily progressive muscle relaxation techniques (Jacobson’s protocol) decreased HR and BP and increased breath hold times after 3 months [36]. One hour weekly yoga sessions (warm up, breathing, meditation, stretching) for 12 weeks did not alter LF or HF HRV but did reduce the LF/HF ratio compared to the control group [45]. However, other 1-hour weekly yoga sessions (5 min Meditation, 40 min Yoga/ Breath, 15 min Mindfulness) did not alter HR after 12 weeks despite HR being lower after each session [49]. Interestingly, the participants had an affinity for yoga and mindfulness, which may have impacted their results [49]. A 2 month chair yoga program did have positive effects on heart rate (decrease), HRV (increased RR interval, increased SDNN, increased PNN50, decreased LF, increased HF, decreased LF/HF ratio) compared to individuals who did not complete chair yoga [60]. Lastly, completing 4-hour sessions of attention-based training (focused attention to transcendental or other mantra-based meditations) 4 times and 20 minute practices (attention to Maranatha phrases) twice per day over 7 weeks decreased HR and increased HRV and sleep durations [39].

Overall, mind-body programs lasting 8–12 weeks may require large variations in commitments. According to the studies in the current scoping review, all intervention types included herein seem efficacious for improving physiological parameters despite difficulty in extrapolating precise comparisons due to variations in programs and outcome methodologies. This is especially problematic for HRV measures, which generally remained unchanged from the 8- to 12-week mind-body programs. These findings bring to question whether HRV parameters are capable of detecting physiological changes from mind-body programs, but also whether the methods being used thus far are appropriate. Since HRV is susceptive to many external and internal factors, more frequent testing throughout the mind-body program (such as seen in [39]) may be more appropriate than simple pre- and post-program timepoints. Considering the busy schedules of healthcare professionals and the challenges of coordinating a mind-body program, the time commitments of mind-body programs must be thought about carefully. To induce physiological changes, there may be a necessary requirement of total density or daily time allotment for mind-body practices throughout the intervention length.

## Limitations and remaining gaps in the literature for future studies

The results gleaned from this scoping review and the limitations reported in the studies included can help to inform future studies seeking to investigate the impacts of mind-body resiliency programs on physiological outcomes in healthcare workers. Next steps require high quality research studies that include an adequate sample size with minimal attrition, a comparable control group, validated measures of neurophysiological metrics, and long term follow up timepoints. Previous research has found mind-body interventions to reduce stress hormones, blood pressure, and heart rate in a range of populations [84], while evidence of mind-body program effects on HRV seem inconclusive at this time [85]. The previous findings are consistent with the current findings of this study, but healthcare workers may have unique schedule complications and occupational pressures to consider. For example, some have found that offering mind-body programs does not fully address stress and wellbeing concerns if a supportive environment is not achieved [86]. Thus, mind-body interventions should be realistic for healthcare workers to integrate into their daily schedules and structured to ensure reproducibility in similar environments. Further, the assessment tools utilized should consider technological constraints in adequately and validly assessing physiological parameters. Assessing physiological parameters throughout and beyond the interventions would prove useful for presenting short-term responses and trajectory of long-term physiological adaptations within the same protocols. Lastly, having a comparable control group and treatment groups for healthy and stressed counterparts are necessary to decipher where the most physiological changes may reside. Collectively, these suggestions can fill the current and previously identified gaps in the current literature: a deeper understanding of both the practical (i.e., perceptions of stress and mental wellbeing) and neurophysiological (i.e., heart rate variability, blood pressure, hormones) metrics, long term follow-up periods with compliance data, and high quality randomized controlled trial studies with adequate sample sizes [87].

## Conclusion

A diverse selection of mind-body resiliency building programs for healthcare workers was assessed in this scoping review. Mind-body programs have been shown to impact subjective measures of health and wellness, such as decreased stress and improved wellbeing, but appear to provide mixed results as it pertains to physiological outcomes. The discrepancies may in part be due to the vast differences in methods used to capture physiological states throughout the programs. Clinical studies often employ multi-lead ECG, while validated single-lead ECG or PPG devices may permit the ability to record resting measures in real-world environments with long term follow up evaluation and deliver biofeedback to participants for increased awareness.

Mind-body practices generally resulted in positive physiological responses immediately after sessions, suggesting participants achieved more relaxed mind-body states. Short term programs including yoga, meditation, biofeedback, or breathwork appeared to be more effective at inducing physiological improvements in healthcare workers that are experiencing high levels of stress or have impaired resting physiology (associated with stress). Longer mindfulness interventions lasting 8–12 weeks included large variations in time commitments but generally improved resting heart rate and blood pressure. However, HRV parameters remained unchanged in several of these studies, which may attest the methods used to evaluate HRV and the population being studied (i.e., current stress levels). There are too many confounding variables that influence HRV, which ultimately may affect the evaluation of only pre- and post-program timepoints. Of note, participants that practiced more mindfulness sessions at home had greater improvements to physiological parameters. Thus, individuals may notice greater changes as they gain more experience. Physiological changes appear to be more prudent when current stress levels are high and the necessary sessions throughout the mind-body program are completed.

## Supporting information

S1 Checklist Preferred Reporting Items for Systematic reviews and Meta-Analyses extension for Scoping Reviews (PRISMA-ScR) Checklist. (PDF)
